# Initial validation of a screening tool for disordered eating in adolescent athletes

**DOI:** 10.1186/s40337-020-00364-7

**Published:** 2021-02-15

**Authors:** Samantha F. Kennedy, Jeffrey Kovan, Emily Werner, Ryley Mancine, Donald Gusfa, Heather Kleiman

**Affiliations:** 1 Department of Psychiatry, Michigan State University, 909 Wilson Rd, Room B119, East Lansing, MI 48824 USA; 2 Department of Sports Medicine, Michigan State University, East Lansing, MI 48824 USA; 3 Department of Kinesiology, Michigan State University, East Lansing, MI 48824 USA; 4 Michigan State University College of Osteopathic Medicine, Michigan State University, East Lansing, MI 48824 USA; 5 Grand Ledge High School, Grand Ledge, MI 48837 USA

**Keywords:** Anorexia nervosa, Bulimia nervosa, Sports, Disordered eating, Eating disorder, Screening tool, Desa-6

## Abstract

**Background:**

Disordered eating (DE) is a growing problem among all athletes, particularly adolescents. To help prevent the progression of DE to a clinical eating disorder (ED), a brief screening tool could offer an efficient method for early identification of DE in athletes and facilitate treatment. The aim of this study is to validate a screening tool for DE that will identify male and female adolescent athletes of all sports and levels of competition who are at risk for DE. The Disordered Eating Screen for Athletes (DESA-6) consists of only 6 items and was designed for use in both male and female athlete populations.

**Methods:**

Validation involved two phases: Phase I consisted of screening high school athletes using the Eating Attitudes Test (EAT-26) and the DESA-6; and Phase II included inviting all high school athletes categorized as “at risk” after screening, plus age- and self-reported gender- matched athletes categorized as not “at risk”, to complete the same surveys a second time along with clinical interview. Validity and regression analyses were used to compare the DESA-6 to the EAT-26 and EDE 17.0D.

**Results:**

When comparing to clinical interview, the DESA-6 had a total sensitivity of 92% and specificity of 85.96%, respectively. Upon comparison of concurrent validity, Phase II DESA-6 had a strong significant positive correlation for both males and females when compared to Phase II EDE 17.0D.

**Conclusions:**

A brief, easy to administer screening tool for recognizing DE that can be used by physicians, psychologists, athletic trainers, registered dietitians, and other sport/healthcare staff is of utmost importance for early intervention, which can lead to improved treatment outcomes. The DESA-6 is a promising tool for risk assessment of DE in athletes.

**Supplementary Information:**

The online version contains supplementary material available at 10.1186/s40337-020-00364-7.

## Plain English

Disordered eating, a subclinical spectrum of eating disorders, can potentially progress into numerous physical health complications. Disordered eating may progress to a clinical eating disorder if left untreated. If individuals with disordered eating are identified quickly, treatment outcomes may improve. Athletes are particularly susceptible to disordered eating. This manuscript attempts to introduce the Disordered Eating Screen for Athletes (DESA-6) screening tool, which aims to quickly identify both males and females who may be at risk for disordered eating. Current screening tools are generally only focused on validation in females, but the DESA-6 also aims to be validated in males. Additionally, the DESA-6 is much shorter than previous screening tools and significantly shorter than the current gold standard clinical interview. This study showed the DESA-6 have promised as a tool that athletic trainers, primary care physicians, and sport/healthcare staff may use to quickly screen a student athlete for disordered eating behaviors.

## Introduction

The Diagnostic and Statistical Manual of Mental Disorders, Fifth Edition (DSM-5) defines eating disorders (EDs) as “the persistent disturbance of eating or eating-related behavior, which results in altered consumption of food and a significant impairment of physical health and/or psychosocial functioning [1].” There are two primary presentations of EDs, anorexia nervosa (AN) and bulimia nervosa (BN), both of which are characterized by an undue influence of body weight and/or shape on self-evaluation [1]. Additional EDs include binge eating disorder (BED) and other specified feeding and eating disorder (OSFED). The prevalence of EDs in the general population is estimated to be less than 1% for AN and 1–2% for BN [2].

Previous researchers have indicated that eating-related issues exist on a spectrum, with the clinically diagnosable DSM-5 EDs at the more extreme end of the spectrum [2]. Within the spectrum of eating-related issues is a condition referred to as “disordered eating” (DE), a term which has been used in research to generally describe the abnormal and potentially harmful eating behaviors that do not meet diagnostic criteria for clinical EDs [3]. Although it is believed that DE lies on the same spectrum of eating-related issues as ED, there is currently no formally accepted definition for DE. Furthermore, previously suggested definitions may not encompass the full spectrum of potential behaviors. An editorial on the continuum of DE defined DE as a variety of abnormal eating behaviors (e.g. restrictive eating, fasting, excessive eating, diet pills, laxatives/enemas, and purging [4]. This article provided a comprehensive list of potential abnormal eating behaviors; however, it did not include potential abnormal time parameter restrictions. In athletes, DE has been defined as a general term that describes the spectrum of abnormal eating behaviors with the goal of achieving or maintaining an unhealthy body weight [3].” However, not all DE behaviors in athletes are intended to achieve a lower weight. Athletes, especially male athletes, may engage in DE behaviors with the intent to increase muscle mass to improve performance [5, 6].

With the previous definitions and associated drawbacks in mind, we propose the following definition for disordered eating: intentional chronic abnormal, unhealthy eating/drinking behaviors that can lead to clinically relevant problems and do not necessarily meet DSM-5 criteria for eating disorders. This can include a number of behaviors including (but not limited to): restrictive eating (including excessive fasting or frequently skipping meals); overeating or binge eating; purging; and/or the use of weight loss supplements (e.g., diet pills, laxatives, diuretics). In an athlete population, DE is often done with the intent to alter body weight, shape, and/or athletic performance. Although eating patterns similar to those with DE can arise from a lack of nutrition knowledge by the athlete, it is important to distinguish those athletes who engage in these behaviors intentionally as having DE. Adolescent athletes in particular are susceptible to unintentional DE behavior, as they likely have little knowledge of optimal food intake and may have little access to sports dietitians or nutritional knowledge.

DE is a growing problem among all athletes, particularly adolescent athletes [3, 4]. Adolescents with a pattern of DE often results in them having low energy availability, electrolyte abnormalities and dehydration, which all negatively impact physical and mental health, as well as athletic performance [7]. DE can lead to injuries that result in more participation days lost and longer recovery periods from injuries [8]. If untreated, DE could progress to a clinical ED, putting these adolescents at an increased risk of ED-related mood, anxiety, impulse-control and substance use disorders [9]. Additionally, EDs have one of the highest mortality rates of all mental disorders [7] and are associated with many potentially life-threatening complications, including cardiac arrhythmias, bone loss, colon hypofunction and kidney failure [10]. In order to potentially prevent the progression of DE to a clinical ED, a brief screening tool could offer an efficient method for identifying DE in athletes, thus facilitating early identification and necessary treatment [11–13].

Prevalence estimates of DE/EDs among adolescent athletes vary widely due to the differing criteria and definitions between research studies; however, a number of studies have indicated the prevalence of DE and EDs is higher among adolescent athletes than in the general population [14, 15]. The National Athletic Trainers’ Association (NATA), the American College of Sports Medicine (ACSM) and the International Olympic Committee (IOC) have released statements supporting regular screening for DE in athletes using a screening tool designed specifically for athletes [7, 16, 17]. However, the NATA Position Statement noted that widely used questionnaires, such as the Eating Attitudes Test (EAT-26), the Eating Disorder Inventory (EDI) and the Eating Disorder Examination Questionnaire (EDE-Q), should be used cautiously as these screening tools have not been validated in athletes [7]. The EDE-Q is a self-report questionnaire based on the Eating Disorder Examination (EDE), which is the gold standard for clinically diagnosing EDs and is currently on version 17, referred to as EDE 17.0D. For athletes, specifically, there are six ED-specific screening tools in existence, with the four most common being the Athletic Milieu Direct Questionnaire (AMDQ), Female Athlete Screening Tool (FAST), Physiologic Screening Test (PST), and the Brief Eating Disorder in Athletes Questionnaire (BEDA-Q) [11, 13, 18, 19].

A review by Knapp et al. discusses reasons the four athlete-related screeners should be used with caution [20]. Although the AMDQ was validated utilizing the EDE-16, the tool was validated only in female collegiate athletes, therefore it is unknown if the AMDQ could be generalized to non-collegiate or recreational athletes. Additionally, the AMDQ evaluates presence of menstruation, thus is cannot be used to screen male athletes [13]. The FAST exhibits a similar issue as the wording is specific to female athletes and it was validated in a population of female collegiate athletes ages 18 to 23 years [18]. Neither of these screeners would be appropriate to use in an adolescent athlete population of mixed sexes. The PST was developed specifically for DE screening [11]; however, it requires physiologic measurements (e.g., body fat percentage, parotid gland evaluation), which require specialized equipment and/or training, thus making this screener difficult to use in the field. Additionally, the PST includes a question about the regularity of menstrual cycles, so it cannot be used in male athletes. Lastly, although the BEDA-Q was validated in female adolescent athletes, it is unknown if it would be valid for all levels of competition or male adolescent athletes [19]. The BEDA-Q was developed using DSM-IV criteria, which included amenorrhea as a diagnostic criterion, so it is unclear if this screening tool could be generalized to include male athletes.

A standardized, brief, efficient screening tool for DE that could be given to a large population of both male and female athletes is necessary [10, 19]. The aim of this study is to validate a screening tool for DE that will identify male and female adolescent athletes of all sports and levels of competition who are at risk for DE. The Disordered Eating Screen for Athletes (DESA-6) consists of only 6 items and was designed specifically for use in both male and female athlete populations. We hypothesize that the DESA-6 will be an efficient screening tool to identify DE in adolescent athletes, and may help decrease the potential for progression to clinical ED.

## Methods

All methods were approved by the university’s Institutional Review Board, where the full protocol can be accessed. After initial generation of the DESA-6 questions, we conducted pilot testing in a population of adult athletes using the EAT-26 as a comparison to the DESA-6. After a final version of the DESA-6 was decided, the validation involved two phases: Phase I, which consisted of screening high school athletes using the EAT-26 and the DESA-6; and Phase II in which we invited all high school athletes categorized as “at risk” after screening, along with age- and self-reported gender- matched athletes categorized as not “at risk”, to complete the surveys a second time with clinical interview in which we used the gold-standard EDE 17.0D for comparison. Participants were recruited from Midwestern United States high schools during the months of January through June of 2019.

### Eating attitudes test (EAT-26)

The EAT-26 is a 26-item self-report instrument that utilizes a 6-point Likert scale ranging from 1 (never) to 6 (always). The EAT-26 is an abbreviated version of a 40-item scale developed by Garner et al. and is a validated psychometric measure that identifies risk for EDs in the general population [21]. The criterion validity of the EAT-26 was evaluated and found to have an overall accuracy of 0.90 [22]. Additionally, the EAT-26 has a high degree of internal reliability with an alpha coefficient of 0.79 in patients with AN and an alpha coefficient of 0.94 comparing patients with AN and normal controls [21].

Although the EAT-26 was validated in the general population and not athletes [21], it was chosen as the external validity comparison because it is a well-accepted tool for detecting EDs and is a relatively brief measure for use in the field. A score of 20 or greater on the EAT-26 indicates risk for ED [22, 23]; however, the purpose of the DESA-6 is to detect DE and not ED, so a different scoring system was needed. A study of female athletes found a mean EAT-26 score of 12.66 was associated with DE [24]; therefore, a score of 12 was chosen for this study to indicate someone being “at risk” for DE. was agreed upon with expert opinion from two sports psychiatrists.

### Eating disorder examination (EDE 17.0D)

The Eating Disorder Examination is the current gold standard for diagnosing EDs [25]. It is a psychometrically validated semi-structured interview. It has been revised numerous times with the aim of maximizing reliability [26]. The EDE 17.0D is based on the diagnostic criteria outlined in the DSM-5 to evaluate for AN, BN, binge eating disorder and unspecified EDs. It consists of 62 items categorized into four subscales (Restraint, Eating Concern, Weight Concern, Shape Concern) and takes up to an hour to complete. Previous work on validity of the EDE has demonstrated the majority of the individual items are able to discriminate between patients with and without EDs [25, 26]. Additionally, inter-rater reliability of all items has been found to be high [25]. The EDE 17.0D was chosen as the Concurrent validity test because it is the current gold standard for diagnosing EDs [25] and no gold standard for DE exists.

### DESA-6 question development

The DESA-6 was designed for this study by a former professional triathlete based on a previous study that surveyed over 1000 triathletes in the United States [27]. A review of literature was conducted on DE/EDs in athletes and a pool of 18 items was created. The initial pool included triathlon-specific questions and “competitive athletes” questions, which applied to all sports. Interviews were then conducted with randomly selected triathletes to assess the items. The questions, along with the EAT-26 for comparison purposes, were distributed randomly to triathletes via an online survey, which was completed by 1033 adult triathletes. The chi square test was used in that previous study to assess the statistical significance of relationships between EAT-26 scores and the “triathlon-specific” and “competitive athlete factors.” Following statistical analysis, the items were narrowed down to six, which all assessed “competitive athlete factors,” [27] meaning no questions were specific to triathletes, thus applicable to all sports.

Each of the final six questions were purposely designed to assess a specific facet of DE unique to athletes (see Supplemental Digital Content 1, DESA-6). As DE/EDs are associated with an increased injury rate, more frequent injuries or more severe injuries may indicate higher risk for DE [8]; therefore, question 1 was intended to assess the frequency and severity of injuries due to the increased risk for injury and increased length of recovery following injury [10]. Question 2 was intended to assess fear of weight gain but was specifically tailored to athletes by asking about periods of time when they cannot train or are not training as intensely, so fear of weight gain may be more pronounced. This reflects the DSM-5 Criteria B of AN, which is an intense fear of gaining weight and/or of becoming fat [1]. Many athletes believe that losing weight results in improved performance [28], and with DE may be unhappy with their weight even if they are normal weight or underweight; therefore, Question 3 evaluates happiness with weight and Question 4 assesses the intensity of dissatisfaction with current weight. These questions are related to the DSM-5 Criteria C of AN, which is a disturbance in the way in which one’s body shape or weight is experienced [1]. Dieting is a risk factor for development of EDs in female elite athletes [16, 29], thus, Question 5 is intended to assess the presence of dieting. Because being told to lose weight by a coach/authority figure was found to be a risk factor for development of EDs [14], Question 6 is intended to evaluate the presence of this pressure. To verify that the wording was appropriate and sufficient for all athletes, the six questions and answer choices were reviewed for face validity and adjusted following interviews with athletes of different sports and experts in the field, including sports psychiatrists, sports psychologists, and registered dietitians. A positive score, which is a score of 3 or greater, on the DESA-6 is intended to indicate risk for DE.

### Pilot testing

A total of 22 randomly selected adult athletes aged 18–19 years old were recruited at a local sports medicine clinic. Individuals who were recruited were selected at random from the patient list of two sports medicine providers. Inclusion criteria was self-identification as an athlete participating in a “sport”, defined as an activity requiring a higher level of physical exertion and/or skill than walking. This convenience sample was used for pilot testing because it was an available, responsive population that could prove valuable for future validation of the DESA-6.

Participants completed both the DESA-6 and EAT-26. None were excluded. There were 11 female athletes and 11 male athletes. Twelve different sports were represented in this sample. Following informed consent, athletes were provided a paper survey that included demographic questions (age, a free-text gender question, sport, number of training hours per week), anthropometric questions (self-reported height and weight), the DESA-6, and the EAT-26. Identifying information of the participants was not collected in order to assure anonymity. Athletes completed the survey in a private room with a trained researcher available outside the room.

### DESA-6 validation

#### Phase I: initial screening in adolescent athletes

Power was calculated a priori analysis using the standard alpha of 0.05, power 0.95, and effect size of 0.3, which gave a suggested sample size of 111 for phase I of research. High school student athletes (ages 12 to 19 years, defined as those participating in a high school sponsored sport) were recruited from 12 different Midwest high schools via emails to administrators and athletic trainers. Student athletes were provided with consent forms by school faculty approximately one week prior to survey administration by a member of lab personnel in order to obtain parental consent. Student athletes aged 18 to 19 years were provided adult consent forms. A convenience sample of 308 student athletes provided consent, and none were excluded. A total of 17 sports were represented, including basketball, baseball, football, etc. (see Table 3 for full list of sports). Student athletes were provided a paper survey that included demographic questions (age, a free-text gender question, sport, number of training hours per week), anthropometric questions (self-reported height and weight), the DESA-6, and the EAT-26.

#### Phase II: clinical interviews

Based on Phase I results, student athletes classified as “at risk” for DE based on EAT-26 scores (score ≥ 12) were invited to attend the clinical interview to determine presence of DE/EDs. A total of 77 student athletes were classified as “at risk” and all were invited to participate in Phase II. 41 of these “at risk” students consented to participate. We then invited age- and self-reported gender- matched athletes classified “not at risk” to serve as a control group. A total of 82 student athletes (*n* = 41 at risk) completed Phase II and none were excluded. A total 15 sports were represented (gymnastics and rugby were not represented versus Phase I).

Student athletes under the age of 18 years came to the interview with a parent/guardian to provide written consent and assent. Adult student athletes completed a written consent form. Prior to the clinical interview, participants completed the DESA-6 and EAT-26 again. Due to scheduling conflicts, as many athletes were actively competing, time between initial completion of the DESA-6 and the EAT-26 ranged from 3 weeks to 12 weeks. Height was verified with a stadiometer (Seca 216, Germany) and weight was verified with a scale (Detecto 758C, Welch City, MO). The private clinical interview utilized the EDE 17.0D, which is the gold standard for diagnosing EDs (2). Interviewers (*n* = 5) were trained to conduct the interviews by a board-certified child and adolescent psychiatrist with extensive experience treating DE/EDs and athletes. This board-certified child and adolescent psychiatrist then tabulated the results of the EDE 17.0D and interpreted the results of the self-administered DESA-6. The interviewers verified that each survey was completely answered before moving on with the next phase of the study, which resulted in no missing data on the DESA-6 or EDE 17.0D tests. No observed adverse effects from performing the DESA-6, EAT-26, or ED 17.0D were noted.

### Statistical analyses

An EDE 17.0D with a positive score on two out of four subscales was considered positive for DE. This accommodates for the subclinical nature of DE in comparison to ED, which is measured with a score of 3 or more subscales [26]. A DESA-6 score of three or more was considered a positive screening due to its ability to achieve strong sensitivity while maintaining an acceptable specificity. As a score of three was used as a cutoff value, there were no indeterminate scores. Validity and regression analyses were used to compare the DESA-6 to the EAT-26 and EDE 17.0D. All regressions were interpreted via guidelines from “Statistics without maths for Psychology”. Phase I data of the DESA-6 were compared to Phase I EAT-26 scores to show concurrent validity. Phase I data of the DESA-6 were compared to Phase II EDE 17.0D scores to show the predictive validity. Phase II data of the DESA-6 were compared to Phase II EDE 17.0D scores to show construct validity. Phases I and II data of the DESA-6 scores were compared using regression analysis to determine test-retest reliability. Receiver Operating Characteristic (ROC) Area Under the Curve (AUC) analysis was then explored to further assess correlation between Phase II EDE 17.0D and DESA-6 scores [30]. Statistics were performed with Excel Data ToolPak Version 2016, Software, SPSS version 25, and MedCalc.org [31].

## Results

Pilot testing showed that the DESA-6 was written at an appropriate reading level as no participants reported confusing regarding the wording of the questions or answer choices. There were no changes made to the DESA-6 following pilot testing.

**Fig. 1:**
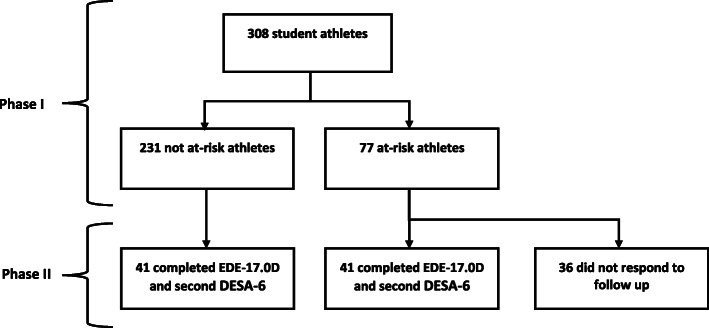
Flow of Participants

Table 1 describes the demographics of athletes recruited in Phases I and II. There were more females than males in Phase II due to more females scoring positively in Phase I. Figure 1 demonstrates the flow of recruitment for Phase I and Phase II of the study.

**Table 1:** Participant Demographics

	Phase I ***N*** = 308	Phase II ***N*** = 82
**Males (n)**	148 (48%)	23 (28%)
**Females (n)**	160 (52%)	59 (72%)
**Mean Age (years)**	15.96	16.17
**Positive DESA-6 Screening Mean Age (years)**	16.14	16.43
**Negative DESA-6 Screening Mean Age (years)**	15.91	16.01
**Mean age and gender adjusted BMI-z score (95% CI)**	0.75 (0.68, 0.82)	0.88 (0.75, 1.03)

Overview of demographics of Phase I and Phase II studies. Scores of ≥ 3 are used as a positive DESA-6 score

Table 2 describes the prevalence found in the adolescent athlete population during Phase I. More female athletes than male athletes were reported due to recruiting variance. Females (Median = 1) were shown to have a significantly higher rate of DE than males (Median = 0) with a Mann-Whitney U test for medians showing U = 7735, *p* < 0.001. This is consistent with populations of previous studies showing higher rates of DE amongst female athletes than male athletes. Total population rates were within the range of previous studies [15]. As participants were being screened for DE behaviors, a clinical spectrum of eating pathologies, other diagnoses are unlikely.

**Table 2:** Prevalence of DE

	Phase I ***N*** = 308		
	Total Sample (***N*** = 308)	Males (***n*** = 148)	Females (***n*** = 160)
**Result**	24.03%	12.84%	34.38%
**CI (95%)**	18.87, 30.16%	7.73, 20.05%	25.59, 44.74%

Prevalence of DE as found by the DESA-6. Scores of ≥ 3 are used as a positive DESA-6 score

Table 3 describes the number of participants who screened positively in Phase I on either the DESA-6 or the Phase I EAT-26. Because athletes were selected at random from a larger population of athletes for participation, this resulted in slightly more females than males and a large discrepancy between the rate of response between each sport. We categorized by sport type, with 5 participants representing aesthetic sports, 234 participants playing ball, 56 from endurance sports, and 13 from weight-dependent sports. The highest number of positive results on either survey were from basketball athletes; however, there was a larger number of basketball athletes surveyed than any other sport.

**Table 3:** Number of Athletes who Screened Positive for DE & ED

Sport Category		Phase I DESA-6		
Type	Sport	Total Sample (***N*** = 308)	Males (***n*** = 148)	Females (***n*** = 160)
**Aesthetic (*n*** **= 5 total)**	Cheer (*n* = 4)	0	0	0
	Gymnastics (*n* = 1)	1	0	1
**Ball (*n*** **= 234 total)**	Basketball (*n* = 56)	14	5	9
	Softball (*n* = 15)	4	0	4
	Soccer (*n* = 31)	9	2	7
	Baseball (*n* = 15)	2	2	0
	Volleyball (*n* = 31)	7	0	7
	Football (*n* = 47)	5	5	0
	Lacrosse (*n* = 17)	7	0	7
	Tennis (*n* = 5)	1	0	1
	Water polo (*n* = 14)	2	0	2
	Rugby (*n* = 2)	0	0	0
	Ice Hockey (*n* = 1)	0	0	0
**Endurance (*n*** **= 56 total)**	Cross Country (*n* = 15)	6	0	6
	Track (*n* = 26)	4	0	4
	Swimming (*n* = 15)	6	0	6
**Weight Dependent (*n*** **= 13 total)**	Wrestling (*n* = 13)	6	5	1

This table gives the number of athletes of each sport who scored positively on the DESA-6 during Phase I. Scores of ≥ 3 are used as a positive DESA-6 score

Additionally, defining “at risk” student athletes as those with a score of 12 or greater on the EAT-26 (versus the typical positive score of 20 or greater), allowed the inclusion of athletes typically considered borderline cases. In Phase II, 41 athletes classified as “at risk” in Phase I consented to complete the EDE 17.0D, with 19 of the “at risk” group screening positive. Of the total 82 Phase II athletes, the DESA-6 screened positive for a total of 31 athletes while the EDE-17D found only 25 to have DE. Table 4 details the comparison of these scores. The DESA-6 favors a positive screen in borderline cases, screening positive for 7 (77.8%) of the borderline positive cases and 7 (75.0%) of the borderline negative cases.

**Table 4:** Overview of DESA-6 Scores in comparison to EDE-17 Subscales

Number of Positive EDE-17 Subscales	Number with EDE-17 Score	Number Screened Positive with DESA-6 (Percent)	Average DESA-6 Score (± 95% CI)
**0**	49	2 (4.1%)	0.71 (0.35, 1.07)
**1**	8	6 (75.0%)	2.63 (1.64, 3.62)
**2**	9	7 (77.8%)	3.00 (1.98, 1.02)
**3**	9	9 (100%)	4.56 (3.88, 5.24)
**4**	7	7 (100%)	5.43 (7.02, 3.84)

Scores of ≥ 2 positive subscales are used as a positive EDE-17 score. Scores of ≥ 3 are used as a positive DESA-6 score

### Diagnostic accuracy of the DESA-6 to the EDE-17 and EAT-26

Table 5 shows the validity analyses for the DESA-6 versus the EAT-26 (Phase I) and versus the EDE 17.0D (Phase II) separated into male and female participants. Compared to the EAT-26 in the total sample analyses, the DESA-6 had a total sample sensitivity of 62.5% and a specificity of 87.7%. When comparing to the EDE 17.0D, the DESA-6 had a total sensitivity of 92% and specificity of 85.96%, respectively. Amongst males there was a sensitivity of 100% and a specificity of 85.0%. Amongst females there was a sensitivity of 90.9% and a specificity of 86.5%. The DESA-6 showed an overall accuracy of 87.80% when compared to the EDE 17.0D. Positive predictive value and negative predictive value are used to compare the rates in which positive and negative screens of the DESA-6 match the EAT-26 or EDE-17.

**Table 5:** DESA-6 Diagnostic Accuracy Measures of the DESA-6 to the EDE-17

**Phase II**		**Total Sample (*n*** **= 82)**	**Males (*n*** **= 23)**	**Females (*n*** **= 59)**
		**Result (95% CI)**	**Result (95% CI)**	**Result (95% CI)**
**DESA-6 vs. EDE-17**	**Sensitivity**	92% (74.0%, 99.0%)	100% (29.24, 100%)	90.9% (70.8, 98.9%)
	**Specificity**	85.96% (74.2, 93.3%)	85.0% (62.1, 96.8%)	86.5% (71.2, 95.5%)
	**Positive Likelihood Ratio**	6.56 (3.65, 14.9)	6.7 (2.35, 18.92)	6.7 (2.95, 15.36)
	**Negative Likelihood Ratio**	0.09 (0.02, 0.35)	0 (0, 0)^b^	0.11 (0.03,0.40)
	**Disease Prevalence**^**a**^ **(EDE-17)**	^a^30.5% (20.8%, 41.6%)	^a^13.0% (2.78, 33.59%)	^a^37.3% (25.04%, 50.%)
	**Positive Predictive Value**	74.2% (60.0%, 84.7%)	50.0% (26.05, 73.95%)	80.0% (63.66, 90.13%)
	**Negative Predictive Value**	96.1% (86.6%, 98.9%)	100% (N/a)^b^	94.1% (80.9, 98.4%)
	**Accuracy**	87.8% (78.7%, 94.0%)	87.0% (66.4, 97.2%)	88.1% (77.1, 95.1%)
**Phase I**		**Total Sample (*n*** **= 308)**	**Males (*n*** **= 148)**	**Females (*n*** **= 160)**
		**Result (95% CI)**	**Result (95% CI)**	**Result (95% CI)**
**DESA-6 vs. EAT-26**	**Sensitivity**	62.50% (50.30, 73.64%)	41.67% (22.11, 63.36%)	72.92% (58.15, 84.72%)
	**Specificity**	87.71% (82.83, 91.61%)	92.74% (86.67, 96.63%)	82.14% (73.78, 88.74%)
	**Positive Likelihood Ratio**	5.09 (3.46, 7.47)	5.74 (2.61, 12.62)	4.08 (2.65, 6.30)
	**Negative Likelihood Ratio**	0.43 (0.32, 0.58)	0.63 (0.45, 0.89)	0.33 (0.21, 0.53)
	**Disease Prevalence (EDE-17)**	23.38% (18.76, 28.51%)	16.22% (10.67, 23.16%)	30.00% (23.02, 37.74%)
	**Positive Predictive Value**	60.81% (51.36, 69.52%)	52.63% (33.58, 70.95%)	63.64% (53.16, 72.96%)
	**Negative Predictive Value**	88.46% (85.00, 91.20%)	89.15% (85.37, 92.04%)	87.62% (81.53, 91.90%)
	**Accuracy**	81.82% (77.05, 85.96%)	84.46% (77.60, 89.89%)	79.38% (72.27, 85.36%)

Overview of the DESA-6’s ability to screen for DE in an athlete population. Scores of ≥ 3 were used as a positive DESA-6 Score. Scores of ≥ 12 were used as a positive EAT-26 Score. For the EDE-17, a positive DE score is defined as a positive score on the “Restraint OR Eating Concern” subscales and a positive score on the “Weight Concern OR Shape Concern” subscale. ^a^ Prevalence is not representative of population due to recruitment constrictions.^b^ Could not be calculated due to no false negative results

**Fig. 2:**
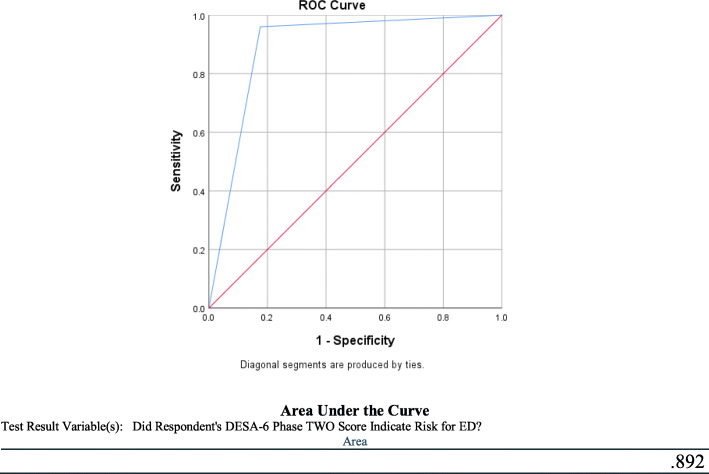
ROC Curve Analysis of the DESA-6 in comparison to the EDE-17.0D

Figure 2 further compares Phase II DESA-6 Scores and EDE 17.0D scores using ROC Curve analysis. This is a calculation of the overall ability of a parameter can distinguish between an experimental and control group. ROC-AUC showed a moderate approaching high correlation of 0.89 between the two measures [32].

### Construct validity

Construct validity of the DESA-6 was performed via regression analysis. Upon comparison of concurrent validity, Phase II DESA-6 had a strong significant positive correlation [*r* (23) = .80, *p* < 0.001] for males and [*r* (59) = .80, *p* < 0.001] for females when compared to Phase II EDE 17.0D. Comparison of the DESA-6 to the EAT-26 showed a low to moderate significant positive correlation with scores of [*r* (146) = .49, *p* < 0.001] in males and a moderate to strong positive correlation of [*r* (160) = .63, *p* < 0.001] in females.

### Reliability

Test-retest reliability comparing DESA-6 scores in Phase I to scores in Phase II showed a strong significant positive correlation [*r* (23) = 0.83, *p* < 0.001] in males and a significant positive correlation in females [*r* (59) = 0.76, *p* < 0.001].

## Discussion

The aim of this study was to validate a brief screening tool to detect risk for DE in adolescent athletes. The DESA-6 showed acceptability in detecting athletes both at risk for DE and athletes without risk for DE with sensitivity of 92% and specificity of 85.96%, respectively, when compared to the gold-standard EDE 17.0D. In comparison, the AMDQ had a sensitivity of 80% and a specificity of 77% [13, 20], the PST had a sensitivity of 86.5% and specificity of 77.7% [11], and the BEDA-Q had a sensitivity of 82.1% and specificity of 84.6% (20). These three screening tools were each created specifically for female collegiate athletes (AMDQ, PST) or female elite athletes (BEDA-Q), so it is worth noting that the DESA-6 had comparable sensitivity and specificity, but in a broader athletic population.

The proportion of adolescent athletes within this study population who were at risk for DE, as determined by the DESA-6, was 30.86%. Two studies conducted in collegiate female athlete populations found 35% of the female athletes had DE [11, 13] and a study by Torstveit et al. in 2008 on adolescent and adult elite female athletes found a prevalence of DE of 32.8% [33]. These prevalence rates are slightly higher than the results of this study, which is likely because our study included both male and female athletes. Although limited research exists on the individual prevalence of DE in female versus male athletes, a study by Sundgot-Borgen and Torstveit using the EDE 16.0 to assess EDs in adolescent and adult female and male elite athletes reported EDs in 20% of female athletes and 8% of male athletes [34]. This study also found a higher prevalence of DE in female athletes with 34.4% of female athletes scoring positive and 12.8% of male athletes scoring positive. The percent of male athletes scoring positive is also similar to a study by Rosendahl et al. in 2009 which found a DE prevalence rate of 10.4% in male high school athletes using the EAT-26 [35].

Although the results of the current study align similarly with previous research, it is important to note that each used different instruments to assess the presence of DE in their population, which contributes to the wide variation in reported rates. Additionally, none of these tools are specific to athletes, which also contributes to the variation.

Previous research has indicated athletes of aesthetic sports (e.g., wrestling, gymnastics) are at higher risk for DE/EDs [34, 36, 37]. In the present study, wrestlers showed the highest prevalence of DE (46%, *n* = 6 of 13, scored positive). A study of DE in Division I college athletes by Engel et al. found elevated levels of drive for thinness, food restriction and purging behaviors in wrestling compared to other sports [38]. Additionally, a study by Chapman and Woodman in 2016 found wrestlers reported a greater incidence of DE [39]. The number of gymnasts in our population was too low (*n* = 1) for further analysis. This is due to the high schools involved in this research, as only one offered gymnastics as a school sport.

Research has also indicated endurance athletes (e.g., cross-country, swimming) are at higher risk. In the present study in Phase II, 40% (*n* = 6 of 15) of cross-country athletes and 40% (*n* = 6 of 15) of swimmers scored positive on the DESA-6. Higher-than-average rates of DE among distance runners and swimmers was expected, as previous research has indicated endurance athletes are at higher risk for DE/EDs [34]. Additionally, a study by Schtscherbyna et al. reported 44.9% of adolescent female elite swimmers had DE [36].

Research has also indicated that risk for DE/EDs is not isolated to endurance or aesthetic sports [2, 11, 13, 18]. A study by Black et al. found DE/EDs among athletes representing 10 of 12 sports studied, including swimming, tennis, track & field, volleyball, cross country, golf, gymnastics, cheer, dance and modern dance [11]. A study by McNulty et al. found female athletes with EDs in 5 different sports, including crew, cross country/track & field, lacrosse, soccer and softball [18]. In the present study, DE was detected in athletes of 14 out of 17 sports represented. The highest prevalence of DE among ball sports was lacrosse (41%, *n* = 7 of 17 scored positive). This above-average prevalence may be due to all lacrosse athletes assessed being female. The only sports in this study without positive DESA-6 scores were cheer, ice hockey and rugby. This is likely due to the fact that the number of athletes assessed in each of these sports was small (*n* = 4, 1 and 2, respectively). Thus, this result does not mean athletes in these sports are not at risk for DE as the sample size was too small for analysis.

### Strengths, limitations, and future directions

Strengths of the study include the fact that the sample size includes male athletes and multiple sport types. The DESA-6 is unique because of the inclusion of male athletes in our populations, which are typically not included in samples for statistical validation of screening tools regarding DE behavior risk. Additionally, the brevity and lack of required physiological measurements makes the DESA-6 easy to administer to athletes of all types. Also, this study was able to assess validity across multiple diagnostic tools. However, this study is not without limitations. First, the DESA-6 was pilot tested in an athlete population at the higher end (ages 18 to 19 years) of the age range of the validation population (ages 12–19 years) due to convenience, thus limiting our ability to interpret results from pilot testing for applicability in the lower end of the validation population age range. Second, as data were collected through self-report surveys, the results may be susceptible to response bias. We attempted to limit response bias as much as possible by having the athletes complete the EAT-26 and DESA-6 in private and assuring the participants that results would not be shared with coaches or their school. Additional response bias could be due to only 41 of 77 “at risk” athletes consenting to complete Phase II. Multiple attempts were made to contact all of the “at risk” athletes. Third, although the DESA-6 was developed based on the DSM-5 criteria for ED and expert opinion, the lack of a universally accepted definition and diagnostic criteria for DE gives room for question of the content validity of the DESA-6. However, the similarity in results between the DESA-6 and other screeners, as well as high sensitivity and specificity based on the gold-standard EDE 17.0D give support for validity of this screener. A further limitation of this study is that current results are specific to adolescent athletes of one geographical area (Midwest USA); therefore, generalizability to athletes of other ages or geographic regions cannot be done at this time. Additionally, participants in phase II of the study were not matched to a control by sport, though they were matched by age and gender. A further limitation may be the variations in length of time between completion of Phase I and Phase II, which unfortunately was due to the complicated practice and competition schedule of the student athletes. Further research aimed at addressing these concerns is necessary, including testing validity of the DESA-6 in adult athletes (ages > 19 years) and younger athletes of different levels of competition, inclusion of genders other than male or female, as well as different regions of the United States.

## Conclusions

The DESA-6 is currently the shortest screening tool for flagging risk of DE in athletes, as well as the only known screening tool that can be used for adolescent athletes aged 13 to 19 years of both genders and all sports. A screening tool which assesses DE in athletes is essential due to the high prevalence of DE/EDs in athletes versus the general population [2, 19]. As all athletes face a unique variety of struggles pertaining to eating and nutritional needs, a tool that successfully accounts for male and female athletes of all sports and all levels of competition is essential. Additionally, the use of screening tools for the early detection of DE in athletes has been recommended to help prevent long term consequences, as well as to provide benefits associated with early intervention. This necessity has been recognized by multiple previous studies, as well as the NATA, IOC, and ACSM [7, 16, 17, 20]. A shorter, more efficient screening tool for DE that could be given to a large athlete population without extensive investment of time and resources is necessary, as no current tools can quickly evaluate a large group of adolescent athletes [10, 19]. With only 6 questions, the DESA-6 can be used by physicians, athletic trainers, registered dietitians, or other sport/healthcare personnel to screen an athlete or an entire team quickly and efficiently (brief administration and scoring duration). The DESA-6 does not require psychometric expertise, an extensive time commitment, nor a large number of resources, thus making it a promising tool for risk assessment of DE in adolescent athletes. Future research investigating the validity of the DESA-6 in additional athlete populations, such as adult athletes and sports that were under-represented in this study, could further expand the generalizability of the DESA-6. This could potentially result in the ability to use the DESA-6 to assess all athletes, no matter age, gender, sport, or level of competition.

## Supplementary Information

**Additional file 1.** Disordered Eating Screening for Athletes: 6 Question Screening Tool.

## Data Availability

The results of this study are presented clearly, honestly, and without fabrication, falsification, or inappropriate data manipulation. Additionally, the results of the present study do not constitute endorsement by ACSM. The datasets generated and analyzed during the current study are available from the corresponding author upon reasonable request.

## Abbreviations

DE: Disordered eating
ED: Eating disorder
AN: Anorexia nervosa
BN: Bulimia nervosa
EAT-26: Eating Attitudes Test
OSFED: Other specified feeding and eating disorder
DESA-6: Disordered Eating Screen for Athletes
DSM-5: Diagnostic and Statistical Manual of Mental Disorders, Fifth Edition
BED: Binge Eating Disorder
NATA: National Athletic Trainers’ Association
ACSM: American College of Sports Medicine
IOC: International Olympic Committee
EDE-Q: Eating Disorder Examination Questionnaire
EDE: Eating Disorder Examination
AMDQ: Athletic Milieu Direct Questionnaire
FAST: Female Athlete Screening Tool
PST: Physiologic Screening Test
BEDA-Q: Brief Eating Disorder in Athletes Questionnaire
