# Supplementary material for: Initial validation of a screening tool for disordered eating in adolescent athletes
Source: J Eat Disord. 2021 Feb 15;9:21. doi: 10.1186/s40337-020-00364-7 (PMC7885388; doi:10.1186/s40337-020-00364-7)
Supplement: Supplementary file 1 — Additional file 1. Disordered Eating Screening for Athletes: 6 Question Screening Tool. [file 40337_2020_364_MOESM1_ESM.pdf]

Disordered Eating Screening for Athletes: 6 Question Screening Tool

Please Fill Out Both Sides Completely

Age in Years: ___________ Primary Sport: ____

Gender: ________________ Current Height: ___________ Current Weight: _______________

Number of Training Hours per Week: ____

DESA-6 Questions

Please circle the answer choice that fits best.

1. Have you had 3 or more injuries in the past season OR did your past season end early due to injury?
    a. Yes
    b. No

2. Do you worry about gaining weight during the off season or when you can't train due to injury?
    a. I worry about gaining weight a few times per week
    b. I worry about gaining weight daily
    c. I worry about gaining weight constantly
    d. I do not worry about gaining weight.

3. Are you happy with your current weight?
    a. Yes
    b. No

4. How many pounds do you think you need to lose to be at your best performance weight?
    a. 1 to 5 pounds
    b. 5 to 10 pounds
    c. 10 to 15 pounds
    d. 15+ pounds
    e. None

5. Do you follow a specific diet plan (low fat, low carbohydrate, low fat, low sugar, high protein, etc.) to achieve your best performance weight?
    a. Yes
    b. No

6. Have you ever been told you should lose weight by someone who is not a health professional, such as a coach, fellow athlete or family member?
    a. Yes
    b. No

EAT-26 Questions

Please check one response for each of the following statements:

	Always:	Usually:	Often:	Some times:	Rarely:	Never:
1. Am terrified about being overweight						
2. Avoid eating when I am hungry.						
3. Find myself preoccupied with food.						
4. Have gone on eating binges where I feel that I may not be able to stop.						
5. Cut my food into small pieces						
6. Aware of the calorie content of food that I eat.						
7. Particularly avoid foods with high carbohydrate content (i.e. bread, rice, potatoes, etc.).						
8. Feel others would prefer if I ate more.						
9. Vomit after I have eaten.						
10. Feel extremely guilty after eating.						
11. Am preoccupied with a desire to be thinner.						
12. Think about burning up calories when I exercise.						
13. Other people think that I am too thin.						
14. Am preoccupied with the thought of having fat on my body.						
15. Take longer than others to eat my meals.						
16. Avoid food with sugar in them.						
17. Eat diet foods.						

18. Feel that food controls my life.						
19. Display self-control around food.						
20. Feel that other pressure me to eat.						
21. Give too much time and thought to food.						
22. Feel uncomfortable after eating sweets.						
23. Engaged in dieting behavior.						
24. Like my stomach to be empty.						
25. Have the impulse to vomit after meals.						
26. Enjoy trying new rich foods.						
